# A dual colour FISH method for routine validation of sexed *Bos taurus* semen

**DOI:** 10.1186/s12917-019-1839-3

**Published:** 2019-04-03

**Authors:** Olavi Reinsalu, Ott Scheler, Ruth Mikelsaar, Aavo-Valdur Mikelsaar, Triin Hallap, Ülle Jaakma, Peeter Padrik, Ants Kavak, Andres Salumets, Ants Kurg

**Affiliations:** 1 Institute of Molecular and Cell Biology, University of Tartu, Riia 23, 51010 Tartu, Estonia; 2 The Competence Centre on Health Technologies, Tiigi 61B, 50410 Tartu, Estonia; 3 Department of Chemistry and Biotechnology, TalTech University, Akadeemia tee 15, 12618 Tallinn, Estonia; 4 Faculty of Medicine, University of Tartu, Ravila 19, 50411 Tartu, Estonia; 5 Institute of Veterinary Medicine and Animal Sciences, Estonian University of Life Sciences, Kreutzwaldi 62, 51014 Tartu, Estonia; 6 Animal Breeders Association of Estonia, Koogimäe 4, Keava, 79005 Kehtna parrish, Estonia; 7 Department of Obstetrics and Gynecology, Institute of Clinical Medicine, University of Tartu, Puusepa 8, 50406 Tartu, Estonia; 8 Department of Biomedicine, Institute of Biomedicine and Translational Medicine, University of Tartu, Ravila 19, 50412 Tartu, Estonia; 9 Department of Obstetrics and Gynecology, University of Helsinki and Helsinki University Hospital, Haartmaninkatu 8, 00014 Helsinki, Finland

**Keywords:** Cattle, Sexing, Sperm, Fluorescence in situ hybridisation

## Abstract

**Background:**

Usage of sexed semen that allows to choose the gender of the calves, is commonly practiced in livestock industry as a profitable breeding alternative, especially in dairy farming. The flow cytometric cell sorting is the only commercially available method for bovine sperm sexing. For validation of the sexing procedure several methods have been developed including sperm fluorescence in situ hybridisation techniques. Latter usually include the use of pre-labelled nucleotides for probe synthesis which is relatively expensive approach compared to combined application of aminoallyl-dUTP and chemical binding of fluorescent dyes. Here a sex determining dual colour bovine sperm fluorescence in situ hybridisation method is presented which is considered more cost-effective technique than the previously reported approaches.

**Results:**

The reliability of sex chromosome identifying probes, designed in silico*,* was proven on bovine metaphase plate chromosomes and through comparison with a commercially available standard method. In the dual colour FISH experiments of unsexed and sexed bovine sperm samples the hybridisation efficiency was at least 98%, whereas the determined sex ratios were not statistically different from the expected. Very few cells carried both of the sex chromosome-specific signals (less than 0.2%).

**Conclusions:**

A protocol for a dual colour bovine sperm FISH method is provided which is cost-effective, simple and fast for sex determination of spermatozoa in bull semen samples.

## Background

With the growing human population the need for all resources rises. Livestock farms, for instance, have to increase the output of beef and milk at least 60% during next 30 years to keep up with the rising food demand [1]. Inevitably, to maintain a cost-effective management of dairy farming one of the key elements is to increase the herd genetic value towards high-producing but more resilient animals. In dairy industry, artificial insemination with sexed semen from genetically valuable bulls allows to receive more female calves with the better genetics in shorter time interval.

The only currently commercially available bovine sperm sorting method is based on flow cytometric fluorescence activated cell sorting (FACS). The X and Y chromosome carrying sperm are separated with 90% accuracy by the difference in the amount of DNA in the sperm head. Although the method is considered relatively expensive and the sexed semen had lesser fertility in the past, recent improvements in FACS have made sexed semen near equally fertile [2]. Thereafter the FACS sorted semen has been more rapidly adapted by the milk and beef industry.

Sexed semen purity is usually verified by reanalysis with flow cytometry [3] but the validation of results, by a reliable method which does not rely on the same instrumentation and criteria, is essential for the practical use. For a routine evaluation of the purity of sexed semen the method has to be both reliable and affordable. The most commonly used molecular techniques for assessing the purity of sexed semen are quantitative polymerase chain-reaction (PCR) [4, 5] and fluorescence in situ hybridisation (FISH) [6–9] since these are simple, accurate, easy to use and cost-effective methods. Notably FISH allows to identify the sex of spermatozoa on a single cell level and also determine the rate of aneuploidies in the analysed semen [10, 11].

FISH analysis for sex determination requires at least one sex chromosome-specific probe, but ideally a probe for each sex chromosome. Due to the nature of DNA probes the size of hybridisation signals depends on the length of the probes [12]. Large bacterial artificial chromosome-derived clones, cosmid clones or degenerate oligonucleotide-primed PCR (DOP-PCR) amplicons from chromosome microdissections are often used as probes to receive intensive FISH signals [13–16]. Alternatively, shorter probes may be used which are complementary to certain chromosome-specific repeats [6, 8, 9].

All of the given reports use direct labelling of DNA FISH probes through PCR in which nucleotides carrying a fluorescent label are incorporated into the probes by DNA polymerase [6–11, 13–16]. Since these fluorescently-labelled nucleotides are much larger molecules, the high risk of steric hindrance can make generation of the probes less productive. In here we show alternative to direct labelling methods in preparing FISH probes by incorporating small modified nucleotide analogues into the probes through PCR. This is followed by chemical labelling reaction with fluorescent dyes. We use 5-[3-aminoallyl]-2′-deoxyuridine-5′-triphosphate (aa-dUTP) that is well-known nucleotide analogue for such labelling purposes [17]. Our approach has higher labelling efficiency and is more cost effective compared to direct labelling methods. The goal of this study was to develop a low-cost, easy to use and fast dual colour FISH method for identifying both sex chromosomes in sexed bovine semen.

## Results

Considering the minimal PSA threshold for detectable FISH signal is 25 pmol/μg [18] the indirect labelling protocol yielded FISH probes with high labelling efficiencies for both Cy^®^3 labelled and Cy^®^5 labelled probes. The PSA level for pooled Cy^®^3 labelled probes ranged from 45 to 70 pmol/μg and 80 to 110 pmol/μg for the Cy^®^5 labelled probes.

**Fig. 1:**
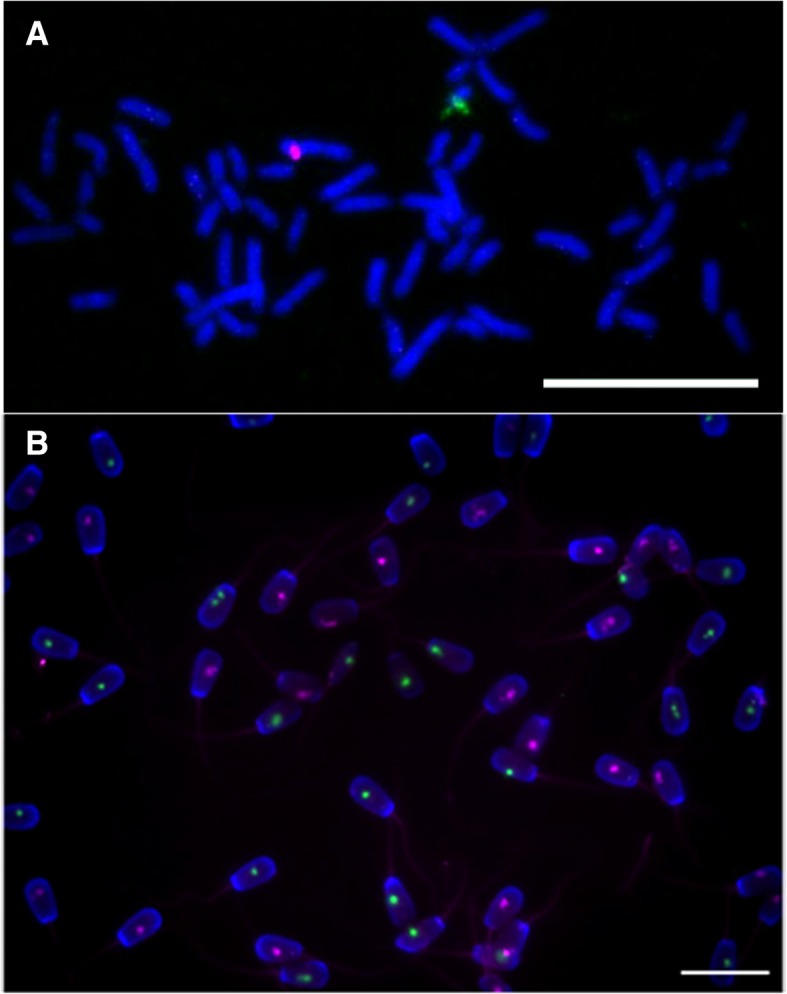
Dual colour FISH experiments with **a**) metaphase plate chromosomes of male bovine blood cells and **b**) native bovine sperm. The images were captured under a 100x magnification immersion-oil objective. The scale bars represent a distance of 20 μm

The dual colour FISH experiments on metaphase plate chromosomes from bull blood cells showed that the probes yield an intensive and well concentrated signal. The Cy^®^3 labelled probes hybridize to a defined region on the X chromosome (Fig. 1a) just as the Cy^®^5 labelled probes hybridize on the Y chromosome, confirming the specificity of the probes. The cells in interphase also carried a concentrated signal of both probes (data not shown).

Likewise to metaphase plates the FISH experiment on unsorted bovine sperm yielded highly intensive and defined signals (Fig. 1b). A total of 1489 spermatozoa were counted whereas almost all of the cells carried only one type of signal (98.7%), very few had both of the signals (0.1%) and some were blank (1.2%) (Table 1). Of the spermatozoa carrying only one type of signal nearly half emitted X chromosome-specific Cy^®^3 signal (50.4%), while the other half bore Y chromosome-specific Cy^®^5 signal. Statistical analysis confirmed that the ratio of cells carrying either signal was not significantly different from the expected 50:50 ratio, the *P*-value of the conducted *χ*^2^ test was 0,7345.

**Table 1:** The results of scoring spermatozoa from dual colour FISH experiments with unsorted and sexed bull sperm

	Unsorted semen			
Type of signal	Cy^®^3	Cy^®^5	Cy^®^3 + Cy^®^5	No signal
Number of cells counted	741	728	2	18
Percentage	49.76	48.89	0.13	1.21
Total number of cells counted	1489			
	Sexed semen			
Type of signal	Cy^®^3	Cy^®^5	Cy^®^3 + Cy^®^5	No signal
Number of cells counted	996	86	2	3
Percentage	91.63	7.91	0.18	0.28
Total number of cells counted	1087			

Compared to the unsorted semen sample even higher hybridisation efficiency was obtained when FISH experiment was performed on sexed semen sample with majority of X chromosome sperms. From 1087 sperm cells counted 99.54% carried one type of signal, 0.18% had both of the signals and 0.28% were blank (Table 1). When comparing the proportion of X-bearing cells (91.63%) with the average proportion stated by the manufacturer (91.4%) of the sexed semen, the difference between the results is statistically insignificant (*P*-value 0.4445) according to *χ*^2^ test.

Frozen-thawed unsorted semen samples were analysed in parallel by FISH method described in this report and a standard FISH method with bovine sex chromosome specific probes. Outcomes of both of the methods did not statistically differ from expected sex ratio of 50:50 (*P*-values 0.0535 and 0.7912, respectively) (Table 2). More importantly, as there is no statistically significant difference between results of the two methods (P-value 0.0991), the probes designed in this study are proven to be reliable.

**Table 2:** The comparison of two FISH methods and P-values of the conducted *χ*^2^ tests. Method A is the method described in this study and method B is a standard method. X and Y chromosome carrying sperm cells were counted

	X	Y
Method A	654 (52.7%)	586 (47.3%)
Method B	718 (50.4%)	708 (49.6%)
	χ2 tests	*P*-values
A vs 50:50	0.0535	
B vs 50:50	0.7912	
A vs B	0.0991	

## Discussion

Flow cytometric bovine sperm sexing technology is increasingly gaining practice by the meat and dairy industry. Through the years several evaluation methods of sperm sexing have already been published, of which FISH-based approaches are most widely used. Interestingly enough, to our knowledge all of the bovine sperm FISH methods use pre-labelled nucleotide analogues to label their probes [6–11, 13–16]. The incorporation of pre-labelled nucleotides is relatively expensive compared to the combination of aa-dUTP and chemical binding of fluorescent dyes. Although a two-step labelling process seems to be more time consuming, the currently reported FISH protocol is swiftly performable especially if the probes are premade and stored in a freezer until needed. The Cy^®^3 and Cy^®^5 signals emitted by the probes are sex chromosome-specific and have high fluorescence intensity, enabling easy distinction between spermatozoa carrying a different sex chromosome.

Compared to Cy^®^3 dye the Cy^®^5 is known to be more susceptible to photobleaching. In addition, both fluorescent dyes have different quantum yield. In order to compensate the sensibility of the Cy^®^5 and dissimilar quantum yield, various nucleotide ratios were used in the first labelling step. For the amplification of Y chromosome-specific probe the relative amount of aa-dUTP was higher (aa-dUTP:dTTP 1:1) compared to the nucleotide mixture for X chromosome-specific probes (aa-dUTP:dTTP 1:2). After labelling with Cy^®^5 and Cy^®^3 dyes, the Cy^®^5 labelled probes bound more dye and emitted more intensive initial signals in FISH compared to Cy^®^3 labelled probes. This way the observation time under fluorescent microscope was prolonged before the Cy^®^5 signal was dimmed out. Cox and Singer [17] labelled human chromosome 17 satellite DNA probes in similar fashion using different aa-dUTP:dTTP ratios and a series of Alexa Fluor^®^ dyes and achieved close labelling efficiencies compared to current report. Their degree of labelling (DOL; 6 dyes per 100 bases) matches with current results when comparing probes with 1:1 aa-dUTP:dTTP ratio (the PSA level range converted to DOL for Cy^®^5 probes is 4–7 dyes per 100 bp).

It is suggested that the purity of template DNA for probe production is crucial [19] as it helps to minimize the amount of probes needed for an optimal signal and the level on background noise. This is even more essential for aneuploidy studies as no unspecificity can be allowed. For bovine sperm X-Y aneuploidy studies two rounds of DNA amplification through DOP-PCR was used in order to receive high quality probes [10, 11]. Products of the first round of the amplification were templates for the second PCR. Likewise, the implication of double amplification in current protocol was considered necessary since it almost completely averted the appearance of background noise.

Through testing of the X chromosome-specific probes it was found that individual use of the probes does not yield any detectable signal. Habermann et al [6] used a mixture of three probes to receive an optimal signal for bovine chromosome 6. The current protocol includes the use of a mixture of five probes to detect bovine X chromosome in FISH observations. Most likely this is a cause of the miniature length of the probes and small number of complementary regions. Short probes bind so much less dye and thus it will not emit a signal bright enough to be detectable. This can be compensated with accumulative effect of higher number of target regions of several probes in order to detect them. On the other hand, the use of several probes to detect a single chromosome seems to cause fragmentation of the signal to some extent. Regardless, this does not prevent from determining a sex chromosome content of a sperm cell.

## Conclusions

A dual colour sex determining FISH protocol is presented which can be used to verify the sex ratio of bovine semen. The reliability of the probes was confirmed by bull blood metaphase chromosome plates and FISH analyses of different types of semen samples. The use of aa-dUTP in a two-step probe labelling method makes this protocol cheaper compared to previously reported FISH based techniques. The provided cost-effective, simple and fast protocol is suitable for a routine validation of sexed bull semen.

## Methods

### Preparation of DNA probes for bovine X chromosome and labelling

To generate bovine X chromosome-specific probes the whole bovine X chromosome genomic sequence (RefSeq accession no. AC_000187.1) from the National Center for Biotechnology Information (NCBI) RefSeq database [20] was BLAST searched [21] for regions complementary to two bovine X chromosome-specific repeats (GenBank accession no. KP677336.1 and AJ884576.1 (both unpublished)). From the regions of interest five pairs of PCR primers were designed, using NCBI Primer-BLAST tool [22], defining five DNA fragments (Table 3).

**Table 3:** The primers used for PCR amplifications of X chromosome-specific probes

	Size of PCR product	Type of primer	Sequence of primer 5′ - > 3’
X1	234 bp	Forward	CTGCTGTGGCTTCCTGGTTA
		Reverse	GTATCATGGCCTCCCTCAGC
X2	532 bp	Forward	GTCAACGGAGGTACAGAGGC
		Reverse	AGCAGACCTCTGGAGACACA
X3	555 bp	Forward	TGGCCAACCAGGAAAAGACT
		Reverse	TGGGACTGCTAATTGTGGGT
X4	203 bp	Forward	CATGAGAAGAAACACCATGCCC
		Reverse	CCACACCCTTCAATCTTGGTCAG
X5	275 bp	Forward	GTCAGTCCTGCAACAGGGAA
		Reverse	TCTGGCACTTTAAATACTGAGAGAC

The PCR amplification of all the X chromosome-specific DNA sequences was carried out in 20 μL reactions consisting of 1X PCR buffer (Solis BioDyne, Tartu, Estonia), 1.5 mM MgCl_2_, 300 μM of each nucleotide (Thermo Scientific, Waltham, MA, USA), 500 nM of each forward and reverse primers (Metabion, Steinkirchen, Germany), 50 ng of bovine genomic DNA and 1.5 U of FIREPol^®^ DNA polymerase (Solis BioDyne). The cycling conditions used were as follows: 15 min at 95 °C, 30 cycles of 5 min at 95 °C, 30 s at 54 °C and 35 s at 72 °C and finalizing 10 min at 72 °C. The PCR products were purified using NucleoSpin^®^ Gel and PCR Clean-up kit (Macherey-Nagel, Düren, Germany) according to manufacturer’s instructions. The amplified DNA samples were pooled.

The indirect labelling protocol was modified from a RNA labelling protocol previously described by us [23]. In the first step of labelling of X chromosome-specific probes aa-dUTP nucleotide analogues were incorporated into the sequences of the probes through multiplex PCR. The 20 μL reaction mix consisted of 1X PCR buffer (Solis BioDyne), 3 mM MgCl_2_, 600 μM of each dATP, dCTP, dGTP, 400 μM of dTTP, 200 μM of aa-dUTP (Thermo Scientific), 500 nM of the each five forward and the five reverse primers (Metabion), 10 ng of the pooled DNA sequences from the first PCR and 1.5 U of FIREPol^®^ DNA polymerase (Solis BioDyne). The cycling conditions used were 15 min at 95 °C, 30 cycles of 5 min at 95 °C, 30 s at 54 °C and 35 s at 72 °C and lastly 10 min at 72 °C. The probes were purified and concentrated until dry using a vacuum concentrator.

The dried DNA samples were resuspended in 4.5 μL of 100 mM carbonate buffer pH 9.0 following addition of Cy^®^3 mono-reactive dye (GE Healthcare, Little Chalfont, UK) dissolved in 4,5 μL of DMSO. The samples were incubated in dark at room temperature for an hour. Excess dye was quenched by mixing with 3.5 μL 4 M NH_2_OH solution after which the labelled probes were separated using NucleoSpin^®^ Gel and PCR Clean-up kit (Macherey-Nagel). The concentrations of DNA and bound dye were measured using a spectrophotometer Nanodrop 2000 (Thermo Scientific). In order to evaluate the efficiency of the labelling a simple calculation method [18], acquiring the probe specific activity (PSA), was implemented, whereas PSA = (pmol of dye per μL) / (μg of DNA per μL). The probe samples were dispensed into aliquots of 1000 ng of DNA and vacuum dried. The dried probes were kept at − 20 °C in dark until use.

### Preparation of DNA probe for bovine Y chromosome and labelling

To generate bovine Y chromosome-specific probe a pair of primers were designed from a bovine Y chromosome-specific repeat sequence btDYZ-1 (GenBank accession no. M26067 [24]). The primers (Table 4), designed using NCBI Primer-BLAST tool, define a 243 bp long DNA fragment.

**Table 4:** The primers used for PCR amplification of Y chromosome-specific probe

	Size of PCR product	Type of primer	Sequence of primer 5′ - > 3’
btDYZ1	243 bp	Forward	TGTAGATGTGTGTGCCATCC
		Reverse	ACCGGTTCCACAGTCTCTAG

The initial amplification of Y chromosome-specific DNA sequence was carried out at the same conditions as for the X chromosome-specific sequences. The incorporation of aa-dUTP through PCR into Y chromosome-specific probe was performed in 20 μL reactions consisting of 1X PCR buffer (Solis BioDyne), 1.5 mM MgCl_2_, 300 μM of each dATP, dCTP, dGTP, 150 μM of each dTTP and aa-dUTP (Thermo Scientific), 500 nM of each forward and reverse primer (Metabion), 10 ng of purified products of initial PCR and 1.5 U of FIREPol^®^ DNA polymerase (Solis BioDyne). The cycling conditions used were as follows: 15 min at 95 °C, 30 cycles of 5 min at 95 °C, 30 s at 54 °C and 35 s at 72 °C and finally 10 min at 72 °C. The probe was purified and concentrated until dry using a vacuum concentrator.

**Fig. 2:**
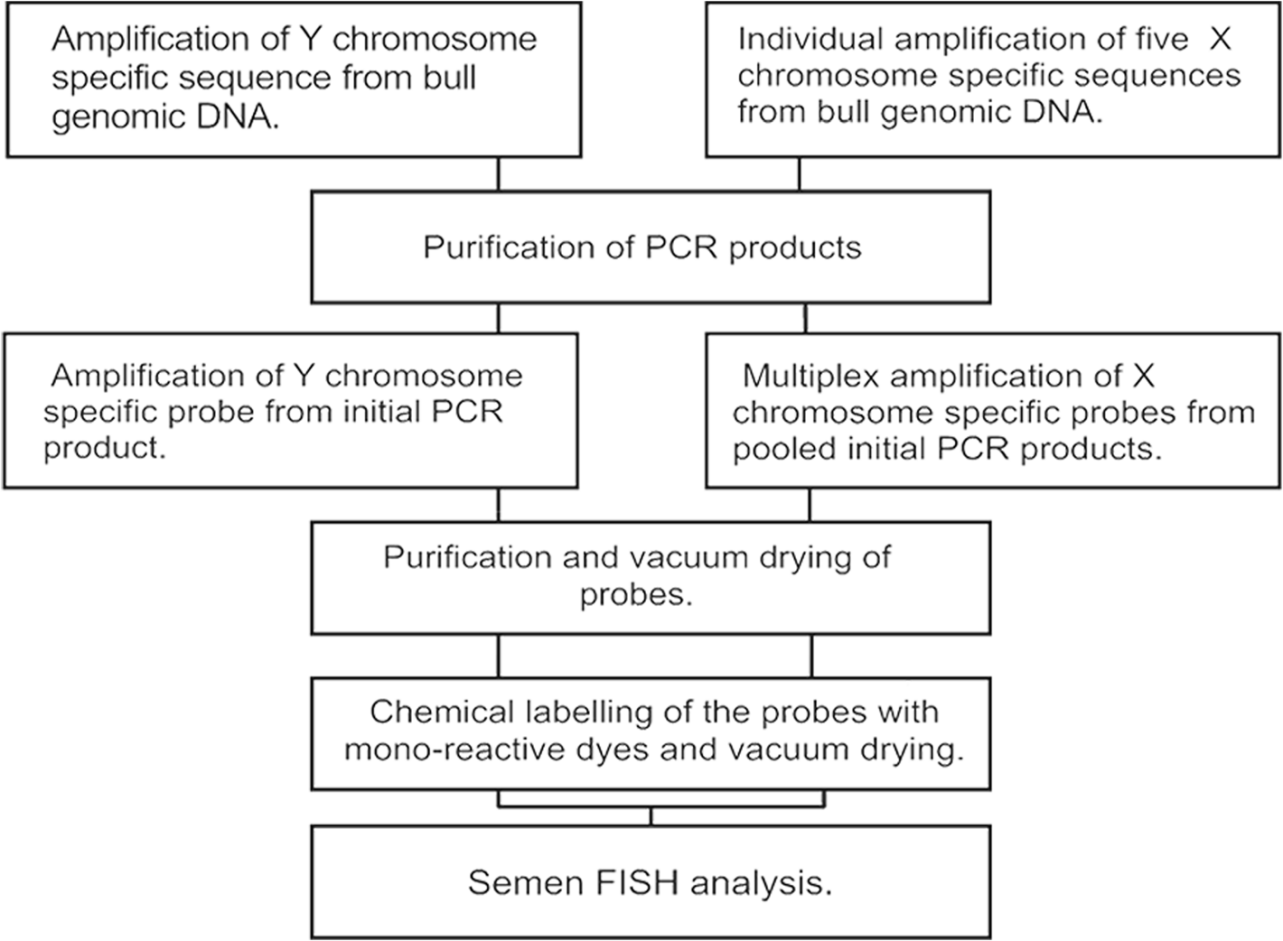
Scheme of sex chromosome-specific probe production. DNA sequences of both type of probes are initially amplified from bull genomic DNA and secondly from the products of the first PCR. After amplification the probes are labelled with mono-reactive dyes and vacuum dried until use

The labelling of the probe was performed as described for X chromosome-specific probes with the exception of the dye used, instead of Cy^®^3 the Y chromosome-specific probes were labelled with Cy^®^5 mono-reactive dye (GE Healthcare). The labelled probe samples were dispensed into aliquots of 500 ng of DNA and vacuum dried. The dried probes were kept at − 20 °C in dark until use. The production of X and Y chromosome specific probes is summarised in Fig. 2.

### Preparation of sperm samples

Commercial unsorted fresh and frozen-thawed semen samples from Estonian Holstein bulls (Animal Breeders Association of Estonia, Keava, Estonia) and a sexed semen sample (Cogent Breeding Ltd., Chester, UK) were prepared by centrifugation through species-specific colloid Bovicoll (J. M. Morrell, SLU, Sweden) according to manufacturer’s instructions. Cleansed sperm was treated with hypo-osmotic KCl solution at 37 °C following fixation with methanol: acetic acid 3:1 solution. Droplets of fixed cells in fixator were dropped and air dried to microscope slides. The slides were kept at − 20 °C until use.

### Dual colour in situ hybridisation on metaphase plates

The FISH probes were validated on metaphase chromosomes from peripheral blood of a normal bull. The slides were incubated 2 min in 2X SSC at room temperature followed by dehydration through incubation in ethanol concentration series (70, 85 and 96%) 1 min each and air-dried. The probes for both chromosomes were suspended in 10 μL of hybridisation solution (50% formamide, 10% dextran sulphate and 1X SSC) and applied to the slides. The slides were covered with a 22 X 22 mm cover slip and sealed with a rubber cement. The DNA was denatured by placing the slides on a heat block at 75 °C for 2 min. The hybridisation of the probes was performed in a dark and moist chamber at 37 °C for 16 h. After hybridisation the slides were washed in stringent 0,1X SSC solution at 62 °C for 5 min two times. The slides were quickly dried in a stream of pressurized N_2_ gas and counterstained with 4′,6-diamidino-2-phenylindole (DAPI).

### Dual colour in situ hybridisation in sperm

The protocol for sperm FISH procedure that was previously described by Habermann et al [6] was adapted with slight modifications. To denature genomic DNA in fixed spermatozoa the slides were immersed in 3 M NaOH for 5 min followed by soaking in four jars of distilled water. The slides were dehydrated in ethanol concentration series (70, 85 and 96%) 2 min each and air-dried. The probes for both chromosomes were suspended in 10 μL of hybridisation solution (50% formamide, 10% dextran sulphate and 1X SSC), incubated at 80 °C for 5 min and chilled on ice until applying to the slides. The slides were covered with a 22 X 22 mm cover slip and sealed with a rubber cement. The hybridisation of the probes was performed in a dark and humid chamber at 37 °C for 2 h. After hybridisation the slides were washed two times in stringent 0,1X SSC solution at 62 °C for 5 min. The slides were quickly dried in a stream of pressurized N_2_ gas and counterstained with DAPI.

### Fluorescence microscopy and scoring

The slides were examined under Olympus BX-61 fluorescence microscope (Olympus, Tokyo, Japan) equipped with phase-contrast optics and a filter set for DAPI, Cy^®^3 and Cy^®^5. Single-channel images were taken with a digital CCD camera XM10 (Olympus) and cellSens Standard software (Olympus). Images in RGB colour were obtained through superimposition of greyscale channels and pseudocolour assignment to them. The spermatozoa carrying either a Cy^®^3 or Cy^®^5 signal, both signals or no signal at all were counted by eye from the images. Overlapping or disrupted cells were omitted. Statistical analyses were conducted using the *χ*^2^ test to compare outcomes with the expected sex ratios.

### Comparison with a standard method

Frozen-thawed semen samples from one bull were analysed in parallel by FISH method introduced in this report and commercially available bovine sex chromosome probes: Bovine IDetect™ Chr X Point Probe Red and Bovine IDetect™ Chr Y Point Probe Green (Empire Genomics LLC, IDLabs, Williamsville, NY, USA). FISH with commercial probes was performed according to the manufacturer’s protocol with slight modification. Images of 15–20 microscopic fields were captured using DP50-CU Photographic system (Olympus) through × 40 objective to count the number of X- or Y-bearing cells. At least 500 spermatozoa were counted for each experiment. The results were statistically compared to expected sex ratio of 50:50 and also between the methods using the *χ*^2^ test.

## Abbreviations

aa-dUTP: 5-[3-aminoallyl]-2′-deoxyuridine-5′-triphosphate
DAPI: 4′,6-diamidino-2-phenylindole
DOL: Degree of labelling
DOP-PCR: Degenerate oligonucleotide-primed polymerase chain reaction
FACS: Fluorescence activated cell sorting
FISH: Fluorescence in situ hybridisation
NCBI: The National Center for Biotechnology Information
PCR: Polymerase chain reaction; PSA: probe specific activity
